# Severe acute respiratory syndrome coronavirus 2 detection by real time polymerase chain reaction using pooling strategy of nasal samples

**DOI:** 10.3389/fmicb.2022.957957

**Published:** 2022-07-22

**Authors:** Annamaria Pratelli, Francesco Pellegrini, Luigi Ceci, Daniela Tatò, Maria Stella Lucente, Loredana Capozzi, Michele Camero, Alessio Buonavoglia

**Affiliations:** ^1^Department of Veterinary Medicine, University Aldo Moro of Bari, Valenzano, Italy; ^2^Clinical Pathology and Microbiology, Hospital Bonomo, Andria, Italy; ^3^Clinical Pathology, Hospital Monsignor Dimiccoli, Barletta, Italy; ^4^Istituto Zooprofilattico Sperimentale di Puglia e Basilicata, Putignano, Italy; ^5^Dental Surgeon, Capurso, Italy

**Keywords:** SARS-CoV-2, real time PCR, nasal swabs, pooling strategy, surveillance

## Abstract

COVID-19 is a life-threatening multisistemic infection caused by *severe acute respiratory syndrome coronavirus 2* (SARS-CoV-2). Infection control relies on timely identification and isolation of infected people who can alberg the virus for up to 14 days, providing important opportunities for undetected transmission. This note describes the application of rRT-PCR test for simpler, faster and less invasive monitoring of SARS-CoV-2 infection using pooling strategy of samples. Seventeen positive patients were provided with sterile dry swabs and asked to self-collected 2 nasal specimens (#NS1 and #NS2). The #NS1 was individually placed in a single tube and the #NS2 was placed in another tube together with 19 NSs collected from 19 negative patients. Both tubes were then tested with conventional molecular rRT-PCR and the strength of pooling nasal testing was compared with the molecular test performed on the single NS of each positive patient. The pooling strategy detected SARS-CoV-2 RNA to a similar extent to the single test, even when Ct value is on average high (Ct 37–38), confirming that test sensibility is not substantially affected even if the pool contains only one low viral load positive sample. Furthermore, the pooling strategy have benefits for SARS-CoV-2 routinary monitoring of groups in regions with a low SARS-CoV-2 prevalence.

## Introduction

Coronavirus disease-2019 (COVID-19) is a life-threatening respiratory and multisystem infection caused by *severe acute respiratory syndrome coronavirus 2* (SARS-CoV-2) and declared pandemic by the World Health organization (WHO) in March, 2020. Virus transmission occurs mainly *via* respiratory droplets released by talking, breathing, coughing, and sneezing, as well as though close contact between people in closed and poorly ventilated environments. Additionally, SARS-CoV-2 can persist in asymptomatic individuals with high titers for up to 14 days, providing important opportunities for silent and undetected transmission (Roque et al., 2021). Therefore, the infection control relies primarily on timely identification and isolation of infected people. In order to limit and circumscribe the pandemic, health authorities around the world urged the development of preventive measures, as virucidal agents (Buonavoglia et al., 2021, 2022) and effective diagnostic tests for rapid and accurate identification of SARS-CoV-2 in the infected patients (Ji et al., 2020), using real-time reverse-transcription polymerase chain reaction (rRT-PCR) on deep nasopharyngeal swabs (NPS) as gold standard test (Azzi et al., 2021). Anyhow, NPS collection is time-consuming, uncomfortable, invasive, can generate stress (chiefly for children), requires trained health care personnel and is associated with a no negligible risk of viral transmission (Azzi et al., 2021). These difficulties were addressed and innovative approaches were proposed for simpler and less invasive sampling and for accelerating the screening of groups of people and large populations (Czumbel et al., 2020; Pratelli et al., 2021). From the very first months of pandemic onset, growing interest was addressed to the use of self-collected sampling as a suitable first-line screening test for SARS-CoV-2 infection (Böger et al., 2020; Mohammadi et al., 2020; Tsang et al., 2021), reducing the risk for healthcare workers involved in sampling, and increasing the number of analyses where supplies of personal protection equipment (PPE) are lacking or difficult to find. In a recent study, Tsang et al. (2021) showed that nasal swab (NS) provides a very good diagnostic performance and that represents a valuable alternative to NPS in the outpatient setting. For the epidemiological monitoring of SARS-CoV-2 infection, especially in “closed” environments (offices, schools, kindergartens, airplanes, etc.), high participation rate and an easy sampling are important concerns to consider when testing asymptomatic population.

The strategy described in the present note represents the application for the diagnosis of SARS-CoV-2 of the pooling test on bovine coronavirus (BCoV) samples reported by Pratelli et al. (2021). The application of an rRT-PCR test for the diagnosis of SARS-CoV-2 using a pooling strategy with 20 NSs, in order to validate the test for systematic mass monitoring of COVID-19 in companies and in schools, is reported.

## Materials and methods

### Study design and sampling

**FIGURE 1:**
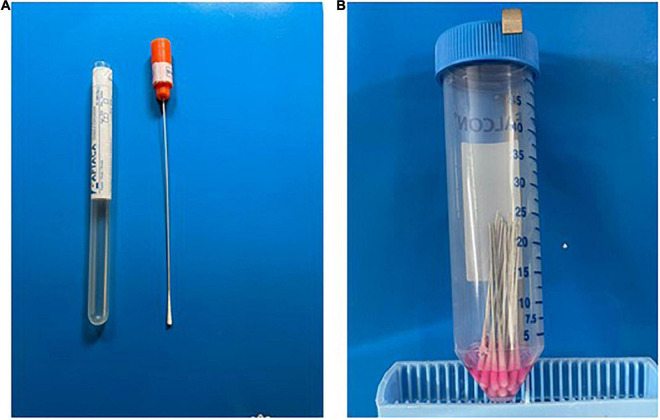
Executive steps of sampling and testing. **(A)** Individual test tube. **(B)** Pool of individual swabs in a 50 mL tube containing 2 mL of DMEM.

The survey was carried out in the laboratory of Clinical Pathology and Microbiology of the hospital Bonomo of Andria, Italy, in collaboration with the hospital Monsignor Dimiccoli of Barletta, Italy, and the Department of Veterinary Medicine of the University of Bari, Italy. The trial was approved and authorized from the Interprovincial Ethical Commission, Area 1, of A.O.U Foggia, ASL FG, ASL BAT (Authorization n°: 34/CE/2022 of February 28, 2022). The COVID-19 positive patients, subject to the release of informed consent, were recruited from March 1st, 2022 to April 1st, 2022 based on the positive rRT-PCR performed on envelope (E), nucleocapside (N), and RdRP genes (Blairon et al., 2021). Specifically, 17 patients individually tested positive were retested using pooling strategy. For this purpose, two sterile dry swabs (Nuova Aptaca srl, Canelli, At, Italy) (Figure 1A) were provided for each positive patient who was asked to self-collected 2 nasal specimens (#NS1 and #NS2) inserting the swabs in the nasal vestibule for up to 1 cm.

### Real-time reverse-transcription polymerase chain reaction

The #NS1 of each positive patient was cut about 5 cm above the cotton swab, individually collected in a single 50 mL sterile conical centrifuge tube and then tested by rRT-PCR. At the same time, the #NS2, after being cut, was collected in a single 50 mL sterile conical centrifuge tube together with 19 NSs collected and cut as described above from 19 patients tested negative with molecular rRT-PCR. Two mL of Dulbecco Minimal Essential Medium (DMEM, Corning, Mediatech, Inc., 9345 Discovery Blvd, Manassa, VA 20109, United States) were added to each conical centrifuge tube containing the pool which included a total of 20 NSs (1 positive NS from each positive patient and 19 negative NSs) (Figure 1B), and to each conical centrifuge tube containing the single positive swab. After vortexing the tubes for 1 min, acid nucleic was extracted using STARMag 96X4 Viral DBA/RNA200C Kit, an automatic nucleic acid purification system with the convenient handling of magnetic beads, and rRT-PCR was set-up using the SARS-CoV-2 assay [Allplex™, Seegene Inc., Taewon Bldg., 91 Ogeum-ro, Songpa-gu, Seoul, Corea Republic) with CFX thermal cycler (Bio-Rad Laboratories S.r.l.Via Cellini, 18/A, 20090 Segrate (MI)—Italy] (Blairon et al., 2021).

## Results

The strength of pooling nasal testing was monitored in each pool of 20 NSs and then compared with the molecular test performed on the single NS of each positive patient. Interestingly, the pooling strategy was able to detected SARS-CoV-2 RNA to a similar extent to the single sample test, even when Ct value is on average high (Ct 37–38), and therefore also in the presence of a low viral load. This is guaranteed by the high sensitivity of the rRT-PCR which allows to identify even a single positive sample within a pool containing up to 19 negative samples. By performing the test on the 20 pooled samples, the difference between the median Ct value of test performed on the single positive NS and the median Ct value obtained on the 20 samples-pool was 3.11, 3.7, and 4.11 Ct, for E, RdRp and N gene, respectively, thus confirming that pooling strategy is at least as sensitive as testing individual samples (Pratelli et al., 2021). This datum confirms that, even if the pool contains only one low viral load positive sample, the sensibility of the test is not substantially affected.

One sample (10B) revealed a low viral load with Ct 34, 35 and 34 in rRT-PCR on single NS for E, RdRp and N genes, respectively, but when tested as pool resulted negative for E and RdRp genes and positive (Ct 37) for N gene.

## Discussion

After more than 2 years the beginning of the SARS-CoV-2 pandemic, epidemiological monitoring and early diagnosis still remain the key factors for the control of the infection. Several antigenic test have been developed, but due to the low sensibility of the assays, rRT-PCR from NPS remains the gold standard for the diagnosis. This sample collection procedure is nevertheless uncomfortable and painful for most people, decreasing the willingness to undergo the test, especially for asymptomatic individuals periodically tested for epidemiological health control (Bergevin et al., 2021).

NSs have been shown to represent a valid alternative sampling method, providing a comparable diagnosis and very good diagnostic performance (Tsang et al., 2021). NSs can be self-collected reducing the health risk of healthcare personnel involved in sampling (preventing any contact with patients) and increasing the number of test that can be carried out especially in regions where PPE supplies are scarce and not very available, and when systematic epidemiological monitoring is required (Ng et al., 2020; Moreno-Contreras et al., 2022). The self-collection for pooled NSs has proven to be a valid and reliable diagnostic method and does not lead to any significant impairment of diagnostic accuracy. The main limits of the study could be the small number of tested patients and, looking forward during the SARS-CoV-2 monitoring in the field, the inadequate sampling by the patient who may not insert the swabs to the proper depth into the nasal vestibules and may not allow sufficient secretions to be adsorbed. Nevertheless, the pooling strategy have practical implications and benefits for SARS-CoV-2 systematic and repeated monitoring.

Similar application has been described (Praharaj et al., 2020; Ayaz et al., 2022). Ayaz et al. (2022) investigated the impact of pool size and mixture level on final Ct values preparing individual swabs and then take an aliquot to create the pool. The advantage of the strategy described in our study lies in the streamlining and simplification of the laboratory procedure for preparing the pool sample and all the 20 swabs will be processed directly to set up the pool, reducing manual work and execution times. Similarly, Praharaj et al. (2020) developed a comparative analysis of pooled samples but testing for only 5- and 10-sample pools.

Pooling of samples allows to increase the number of samples to test, saving the reagent and consequently reducing the costs, especially in developing regions and with a low prevalence of the virus (Abdalhamid et al., 2020; Moreno-Contreras et al., 2022). Pool testing allows to test up to 20 patients with a molecular test, combining the advantage of testing multiple samples with the sensitivity of the rRT-PCR (Garg et al., 2020; Bish et al., 2021). Subsequently, if the pool tested negative, all samples are considered to be below the detection limit of the assay, and no further investigation should be performed. On the contrary, when the pool is positive, the samples must be tested individually to identify the infected patient(s).

The approach of testing pooled samples is cost-effective and useful to decrease the costs of laboratory analyses, scaling and speeding up the monitoring and the epidemiological activities of local or national health authorities, and/or enabling alternative control measures. This is particularly desirable in low-income countries, where limited economic resources can compromise the activation of epidemiological monitoring surveillance plans. Furthermore, systematic screening of medical and paramedical personnel in hospitals and health facilities, as well as of school and companies’ personnel, is a necessity control measure to mitigate and to limit SARS-CoV-2 spreading.

## Conclusion

In conclusion, the pooling strategy on self-collection of samples and rRT-PCR on pooled NSs, was not associated with any significant impairment of diagnostic accurancy.

## Data availability statement

The raw data supporting the conclusions of this article will be made available by the authors, without undue reservation.

## Ethics statement

The study involving human participants was reviewed and approved by the Interprovincial Ethical Commission, Area 1, of A.O.U Foggia, ASL FG, ASL BAT (Authorization n_: 34/CE/2022 of February 28, 2022). The patients/participants provided their written informed consent to participate in this study.

## Author contributions

AB was responsible for the concept. AP was responsible for preparing the manuscript. FP and MC were responsible for the literature review. DT, MSL, LCe, and LCa were responsible for the laboratory analysis. FP and AB critically revised the article. All authors reviewed the manuscript prior to submission and contributed to the article and approved the submitted version.

## Conflict of interest

The authors declare that the research was conducted in the absence of any commercial or financial relationships that could be construed as a potential conflict of interest.

## Publisher’s note

All claims expressed in this article are solely those of the authors and do not necessarily represent those of their affiliated organizations, or those of the publisher, the editors and the reviewers. Any product that may be evaluated in this article, or claim that may be made by its manufacturer, is not guaranteed or endorsed by the publisher.
